# The Landscape of DNA Methylation Associated With the Transcriptomic Network of Intramuscular Adipocytes Generates Insight Into Intramuscular Fat Deposition in Chicken

**DOI:** 10.3389/fcell.2020.00206

**Published:** 2020-04-02

**Authors:** Meng Zhang, Donghua Li, Yanhui Zhai, Zhengzhu Wang, Xiangfei Ma, Daoyu Zhang, Guoxi Li, Ruili Han, Ruirui Jiang, Zhuanjian Li, Xiangtao Kang, Guirong Sun

**Affiliations:** ^1^College of Animal Science and Veterinary Medicine, Henan Agricultural University, Zhengzhou, China; ^2^The First Clinical Hospital, Jilin University, Changchun, China

**Keywords:** DNA methylation, transcriptome, intramuscular adipocytes differentiation, COL6A1, IMF deposition

## Abstract

Intramuscular fat (IMF), which regulated by genetics, nutrition and environment is an important factor that influencing meat quality. Up to now, the epigenetic regulation mechanism underlying poultry IMF deposition remains poorly understood. Here, we focused on the DNA methylation, which usually regulate genes in transcription level. To look into the essential role of DNA methylation on the IMF deposition, chicken intramuscular preadipocytes were isolated and cultured *in vitro*, and a model of intramuscular adipocyte differentiation was constructed. Combined the whole genome bisulfite sequencing (WGBS) and RNA-Seq technologies, we identified several methylated genes, which mainly affecting fatty acid metabolism and muscle development. Furthermore, we reported that DNA methylation regulate intramuscular adipogenesis by regulating the genes, such as collagen, type VI, alpha 1 (*COL6A1*) thus affecting IMF deposition. Overexpression of *COL6A1* increases the lipid droplet and inhibits cell proliferation by regulating *CHAD* and *CAMK2* in intramuscular adipocytes, while knockdown of *COL6A1* shows the opposite effect. Taken together, our results reveal that DNA methylation plays an important role in poultry IMF deposition.

## Introduction

Intramuscular fat (IMF) is one of the most important factors that affect meat quality (Fanatico et al., 2007; Ros-Freixedes et al., 2014; Li et al., 2019). Previous researches have indicated that IMF improved the quality of meat by improving the flavor, juiciness and tenderness (Gao and Zhao, 2009). IMF deposition is primarily dependent on the differentiation, maturation and proliferation of intramuscular preadipocytes (Cristancho and Lazar, 2011; Zhang et al., 2019). Previous studies have identified about several genes related to chicken IMF, including *PPARG*, *GPAT1*, *ACC*, *CD36*, *AGPAT1*, and *DGAT2*, *FABP*, *LPL*, *DGAT1*, and *SCL27A1* (Ye et al., 2010; Serão et al., 2011; Jeong et al., 2012; Li et al., 2013; Qiu et al., 2017). The mechanism that underlies chicken IMF deposition is very complicated obviously, involving many metabolic pathways and genes.

As one of the earliest discovered epigenetic modification, DNA methylation plays an extremely significant role in sustaining cell’s normal function in animals, gene expression regulation (Razin and Cedar, 1984), genetic imprinting (Jaenisch, 1997), embryonic development (Li et al., 2018), and tumor formation (Shivapurkar et al., 1986; Bender et al., 1998). Growing number of studies suggested that DNA methylation played significantly role in adipogenesis (Broholm et al., 2016; Chen et al., 2016; Lim et al., 2016). Previous studies recommended that *DNMT3A* inhibited porcine intramuscular preadipocytes differentiation by changing the methylation levels of *p21* and *PPAR*γ (Abdalla et al., 2018; Qimuge et al., 2019). Zhang et al. (2014) found that *MBD4* inhibited porcine preadipocytes differentiation by changing the DNA methylation levels of adipogenic genes. Li et al. suggested that DNA methylation regulated chicken *PPARG* and *CEBPA* during the development of chicken adipose tissue (Sun et al., 2014; Gao et al., 2015). Our previous study identified large amount of differentially expressed genes (DEGs) during intramuscular adipogenic differentiation (Zhang et al., 2019). The epigenetic molecular mechanism, especially DNA methylation that underlies IMF deposition remains, however, poorly investigated.

In order to investigate the potential functions of DNA methylation that affected the poultry intramuscular adipogenesis. Whole genome single-base DNA methylation profiles of intramuscular preadipocytes and differentiated adipocytes were generated by whole genome bisulfite sequencing (WGBS). The present study integrated the RNA-Seq and WGBS data, aimed to describe the DNA methylation patterns in chicken intramuscular adipocytes and reveal the novel methylated candidate genes related to intramuscular adipogenesis. Our results offered basic research data about intramuscular adipogenesis and the IMF deposition in poultry.

## Results

### The Identification of Chicken Intramuscular Adipocyte Differentiation Model

**FIGURE 1:**
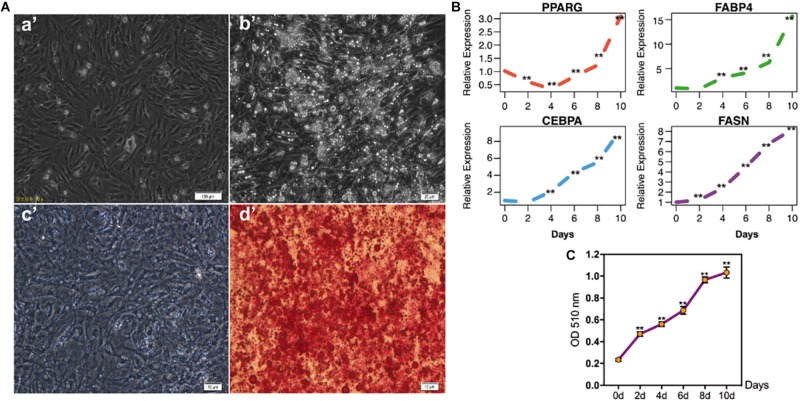
The identification of chicken intramuscular preadipocytes differentiation model. **(A)** The shape of chicken intramuscular preadipocytes before (a’) and after (b’) adipogenic differentiation for 10 days. The oil red O staining of chicken intramuscular preadipocytes (c’) and mature adipocytes (d’); **(B)** qRT-PCR analysis of adipogenic makers PPARG, FABP4, CEBPA, and FASN during chicken intramuscular preadipocyte differentiation. The mRNA levels of adipogenic makers were detected by qRT-PCR at 0, 2, 4, 6, 8, 10 days after induced differentiation. **(C)** The OD value at 510 nm of Oil Red O staining during intramuscular preadipocyte differentiation. (*n* = 3, **p < 0.01).

To investigate the IMF deposition of poultry, chicken intramuscular adipogenic differentiation model *in vitro* was constructed in the present study. After 80–90% confluence, cells were exposed to MDIO differentiation medium. As shown in Figure 1A, cells were filled with lipid droplets after 10 days’ induction. Furthermore, qRT-PCR results suggested that the adipogenic markers *PPARG*, *FABP4*, *CEBPA*, and *FASN* significantly increased with adipogenic differentiation (*p* < 0.01) (Figure 1B).

### Difference in DNA Methylation Level Between Intramuscular Preadipocytes and Adipocytes in Chickens

**FIGURE 2:**
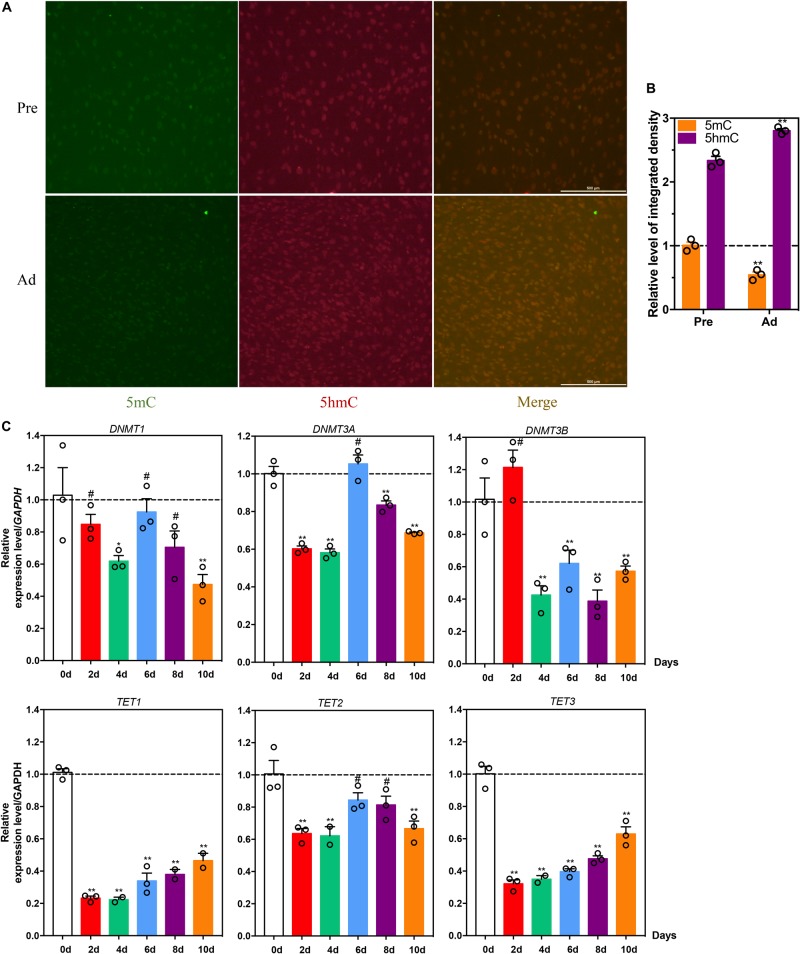
Difference in DNA methylation levels between intramuscular preadipocytes and adipocytes in chickens. **(A)** Immunofluorescence staining and quantification **(B)** of 5 mC (green) and 5 hmC (red) abundance in intramuscular preadipocytes and mature adipocytes. **(C)** Relative mRNA abundance of TET1/2/3, DNMT1, DNMT3A, and DNMT3B during the intramuscular adipogenic differentiation. qRT-PCR analysis of the relative mRNA levels DNA methylasferase DNMT1, DNMT3A/3B, and TET1/2/3 during chicken intramuscular preadipocyte differentiation. (*n* = 3, **p < 0.01, ^#^*p* > 0.05).

To explore the role of DNA methylation in intramuscular adipogenic differentiation, 5 mC and 5 hmC levels were detected by immunofluorescence staining. Compared with intramuscular preadipocytes, the 5 mC levels of intramuscular adipocytes were significantly decreased (Figures 2A,B), whereas 5 hmC levels were higher in the intramuscular adipocytes compared to intramuscular preadipocytes (Figures 2A,B). At the same time, the mRNA expression levels of DNA methylation-related enzymes showed that mRNA expression levels of DNA methyltransferases *DNMT3A*/*3B* and *DNMT1* were significantly decreased after induction of differentiation (*p* < 0.01, Figure 2C), while the demethylase *TET1*/2/*3* were significantly increased after induction of differentiation (from days 2 to 4) (Figure 2C).

### The DNA Methylation Atlas of Intramuscular Preadipocytes and Adipocytes in Chickens

**FIGURE 3:**
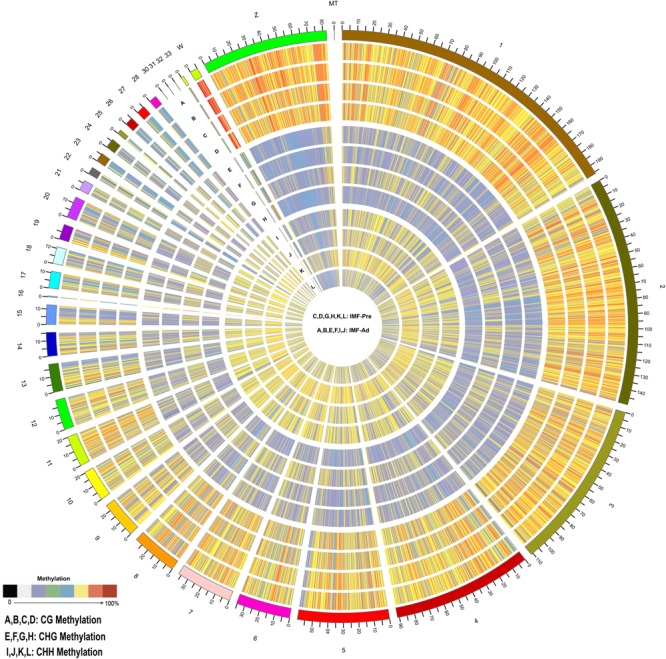
Distribution of identified methylation sites on each chromosome. The outer ring represents the chicken genome labeled with chromosome number and position. **(A–D)** CG Methylation; **(E–H)** CHG Methylation; **(I,J,M,S)** CHH Methylation. **(C,D,G,H,K,L)** IMF_Pre; **(A,B,E,F,I,J)** IMF_Ad.

In the present study, 34.43 and 35.29 G raw data were generated in intramuscular preadipocytes and matured intramuscular adipocytes, respectively. After taking the low quality, N (unknown) and connector contamination reads off, we finally got 212,981,499 and 232,403,717 clean reads in IM_Pre and IM_Ad groups, respectively (Table 1). There were 68.6 and 72% of chicken genome were covered with the uniquely mapped reads in the preadipocytes and adipocytes groups, respectively (Table 1). The unique alignments rate of was more than 80%. The Q30 value was more than 0.9, these results indicated a reliable sequencing outcome. In addition, Circos plot displayed the DNA methylation levels in the various sequence contexts (mCG, mCHG, and mCHH) (where H is A, C, or T) in chicken chromosomes (1–32 and the Z, W, MT chromosome; Figure 3).

**TABLE 1:** The summary of data generated by genome-wide bisulfite sequencing.

					**Unique**	
**Sample**			**Clean**	**Mapping**	**alignments**	
**ID**	**Raw reads**	**Clean reads**	**rate (%)**	**rate (%)**	**rate (%)**	**Q30**
IM_Pre1	376,952,718	347,396,358	92.20	68.60	83.8	0.90
IM_Pre2	293,867,266	276,661,137	94.10	68.60	84.5	0.91
IM_Ad1	359,297,694	330,787,356	92.10	71.70	80.3	0.90
IM-Ad2	329,939,770	316,350,010	95.90	65.80	84.6	0.91

### Global DNA Methylation Patterns Intramuscular Adipocytes in Chickens

**FIGURE 4:**
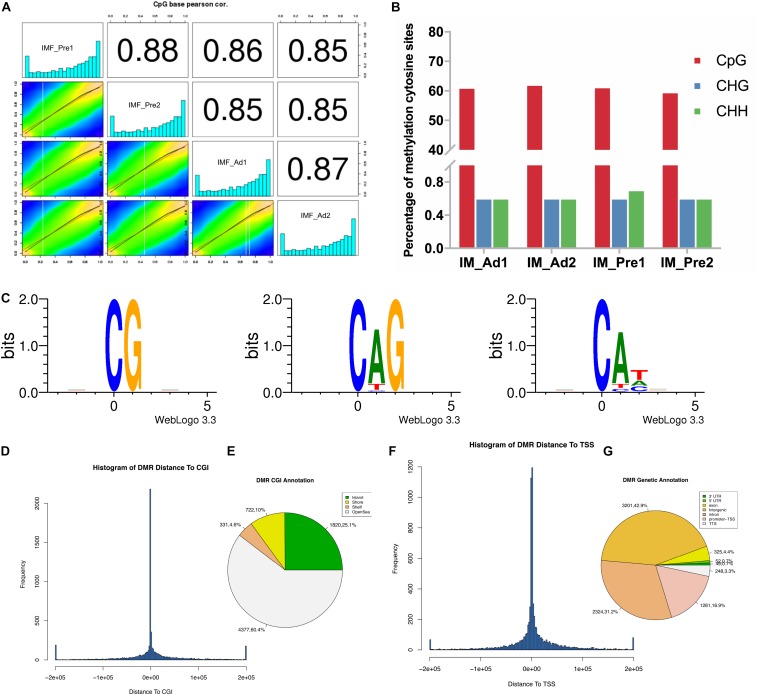
The DNA methylation characteristics of intramuscular preadipocytes and adipocytes in chickens. **(A)** The correlation analysis of the methylation between samples. Heat maps showed the distribution of the methylated CpG sites, the bar plots showed the frequency of methylated CpG sites. **(B)** Comparison of DNA methylation patterns in different samples. **(C)** Sequence preferences for methylation in various sequence contexts. 9 bp base information around the position of mCG, mCHG, mCHH at the highest or lowest methylation levels, in which the methylated cytosine is in the fourth position. **(D)** The frequency distribution histogram of the distance from DMR to CGI. **(E)** The DMR Annotation in CGI functional elements (Island, Shore, Shelf and OpenSea). **(F)** The frequency distribution histogram of the distance from DMR to TSS. **(G)** The DMR Annotation in genome functional regions (5′UTR, 3′UTR, Exon, Intergenic, Intron, Promoter-TSS, and TSS).

Pearson correlation analysis of the CpG base suggested that our samples have good data repeatability (*r* > 0.87) (Figure 4A). To investigate the differences of global DNA methylation profile between the two groups, DNA methylation levels in three contexts: CG, CHG, and CHH (where H is A, C, or T) were analyzed in the present study. As shown in Figure 4B, most proportion (60%) of cytosines were methylated in CpG context, only small proportion (1.2%) of cytosines were methylated in non-CG context (CHG and CHH context). To explore the patterns of methylated cytosines in chicken intramuscular adipocytes, we analyzed the genome-wide mC sequence preferences in various sequence contexts. Our results showed that the methylated cytosines preference for being located in CG, CHG, and CHH (H = A > T) (Figure 4C). The DMRs of the CGI were mainly located in the openSea (60.4%) and CpG island (CGI) (25.1%) (Figures 4D,E). The DMRs were mainly located in the intergenic region (42.9%), followed by the introns (31.25%) and the TSS region (16.9%) (Figures 4F,G).

### Functional Characterization of Differentially Methylated Genes (DMGs)

**FIGURE 5:**
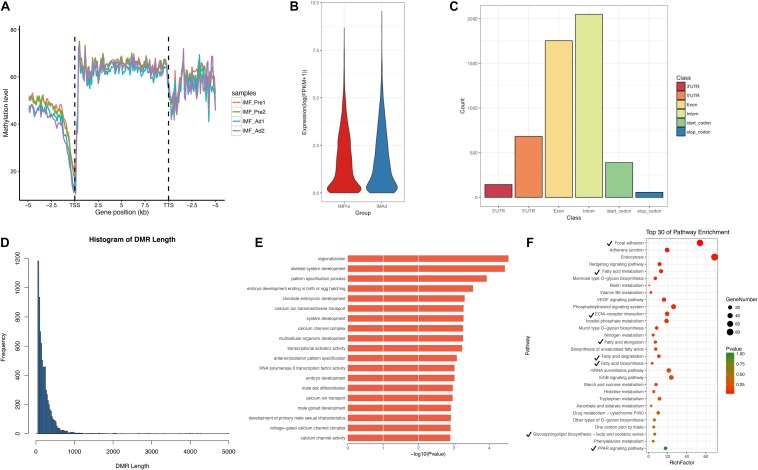
The characteristics of DMRs and function analysis. **(A)** Global DNA methylation levels in different functional regions between different samples. **(B)** Gene expression levels (FPKM) in intramuscular preadipocytes and adipocytes. **(C)** The distribution of DMRs in different functional regions (5′UTR, 3′UTR, Exon, Intron, Start-codon, and Stop-codon). **(D)** The frequency distribution of DMRs count in length. **(E)** GO terms enriched analysis of DMGs. **(F)** Scatter plot of the top 30 KEGG enrichments analysis. Tick represents the pathway involved in lipid metabolism.

In the present study, a total of 7580 DMRs were discovered. The DNA methylation level of adipocytes in the chicken genome showing a “V” trend around the promoter region (Figure 5A), which is consistent with previous studies in chicken breast muscle tissues (Zhang et al., 2017). Furthermore, we found that hypomethylation level in the promoter region and higher genome-wide gene expression level in intramuscular adipocytes groups compared with the preadipocytes group (Figures 5A,B). In addition, a large proportion of DMRs were intron and exon regions (Figure 5C). We noticed that most DMRs were length 100–200 bp and short than 1000 bp (Figure 5D). To look into the DMGs’ potential biological roles, gene ontogeny (GO) analysis and KEGG pathway analysis were performed. Our results showed that the DMGs mainly enriched in the regionalization and skeletal system development terms (Figure 5E), focal adhesion, fatty acid metabolism, ECM-receptor interaction and PPAR signaling pathways (Figure 5F).

### Candidate DMGs Associated With IMF Deposition

**FIGURE 6:**
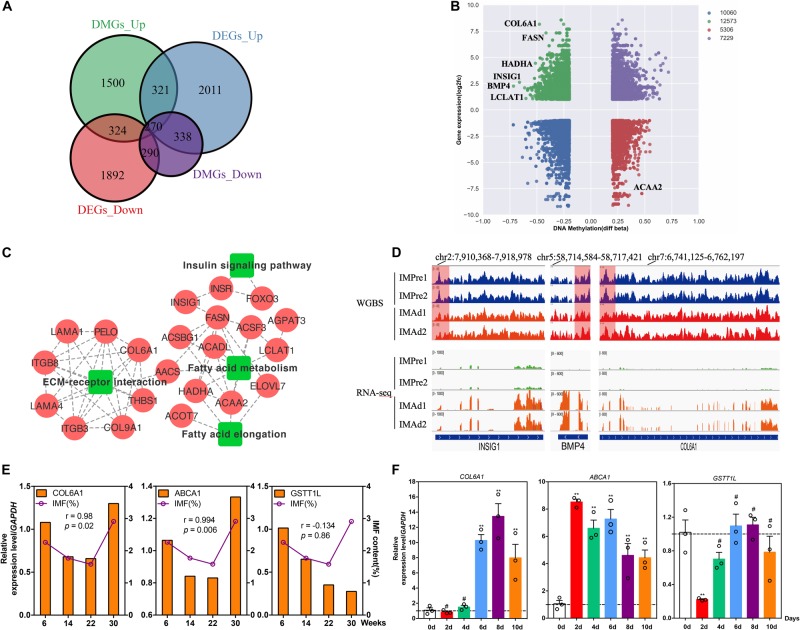
Candidate DMGs associated with IMF deposition. **(A)** The differentially expressed genes (DEGs) overlapped with differentially methylated gene (DMGs) in different groups. **(B)** Integrated analysis of DNA methylation levels and gene expression levels. **(C)** KEGG pathways and protein-protein interaction (PPI) network analysis of candidate DMGs associated with IMF deposition. **(D)** The DNA methylation levels (WGBS) and gene expression levels (RNA-seq) (IGV tracts) of three candidate DMGs (INSIG1, BMP4, and COL6A1). **(E)** The relative mRNA levels of three genes (COL6A1, ABCA1, and GSTT1L) and IMF content in breast muscle at 6, 14, 22, and 30 weeks old. (r, pearson correlation coefficient). **(F)** The relative mRNA levels of three genes during intramuscular adipogenic differentiation (*n* = 3, **p < 0.01, ^#^*p* > 0.05).

To explore whether the candidate DMGs are related to IMF deposition, we integrated the RNA-Seq and WGBS data to reveal methylated candidate genes associated with IMF deposition. Our results showed that there were 324 (hypermethylated and down-regulated) and 338 (hypomethylated and up-regulated) differentially expressed DMGs during adipocytes differentiation process (Figure 6A), several lipid metabolism-related and adipogenic differentiation genes, such as *FASN*, *HADHA*, *INSIG1*, *BMP4*, and *LCLAT1* were found in the present study (Figure 6B). Besides, we observed that several genes were involved in the ECM-receptor interaction, insulin signaling pathway and fatty acid metabolism pathway, such as *COL6A1*, *THBS1*, *LAMA2*, *HADHA*, *ACAA2*, *ELOVL7*, *ACADL*, *LCLAT1*, *INSIG1*, and *FOXO3* (Figure 6C). Moreover, the protein-protein interaction (PPI) network analysis illustrated that these DMGs were correlated with each other highly (Figure 6C). The DNA methylation and gene expression levels of three DMGs, *INSIG1*, *BMP4*, and *COL6A1* were showed in Figure 6D. Furthermore, the correlations between IMF content and gene mRNA levels at different age stages were analyzed. Our results suggested that the expression levels of *COL6A1* and *ABCA1* were positively correlated with the IMF content (*r* = 0.980 and 0.994, *p* < 0.05) (Figure 6E). To study the expression trend of candidate genes in the differentiation of intramuscular adipocytes, the total RNA of intramuscular adipocytes differentiated at different periods was analyzed by qRT-PCR. Our results suggested that the mRNA level of *COL6A1* was significantly increased during adipogenic differentiation of intramuscular preadipocytes. The mRNA level of *ABCA1* significantly increased in the day 2, while declined slowly from days 4 to 10. And *GSTT1L* mRNA expression level was downregulated in day 2, while increased slowly after from days 4 to 10 (Figure 6F). Furthermore, our results suggested that the mRNA level of *COL6A1* was significantly positive correlative with the TG content of intramuscular adipocytes during differentiation process (*r* = 0.84, *p* = 0.03), while *ABCA1* and *GSTT1L* were was not significant correlative with the TG content (*r* = 0.14, *p* = 0.78 and *r* = 0.24, *p* = 0.65) (Supplementary Figure S1).

### DNA Methylation of COL6A1 Promoter Region

**FIGURE 7:**
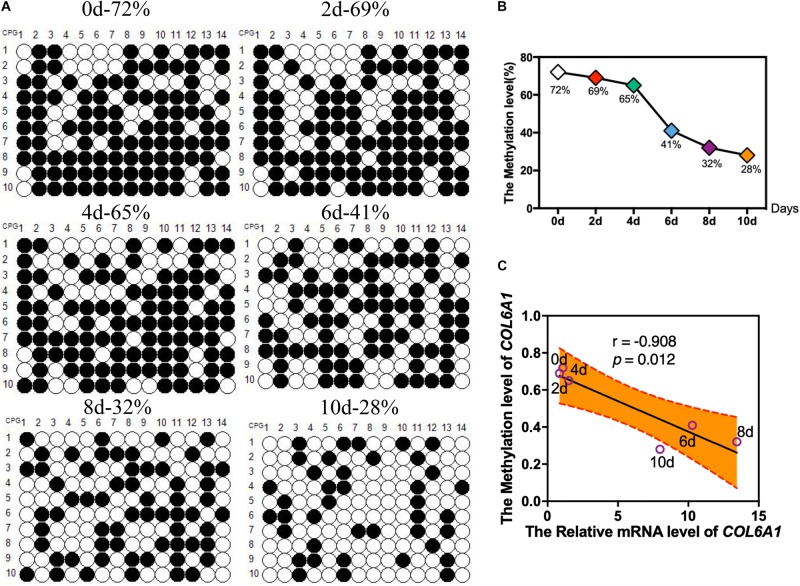
The DNA Methylation levels of COL6A1 promoter region. **(A,B)** The DNA Methylation levels of COL6A1 promoter region in intramuscular preadipocytes and adipocytes. BSP analyses of the DNA methylation of COL6A1 promoter during intramuscular adipogenic differentiation. **(C)** The correlation between the COL6A1 mRNA levels and DNA methylation levels during intramuscular adipogenic differentiation.

According to the BSP results, there was a hypermethylated (72%) promoter region of *COL6A1* in the intramuscular preadipocytes compared with differentiated adipocytes, while a low methylation level (28%) in the matured intramuscular adipocytes (Figures 7A,B). Furthermore, we found that the methylation of *COL6A1* promoter were significantly negatively correlated with the mRNA level (*r* = −0.908, *p* < 0.05) (Figure 7C). And the DNA methylation levels of *ABCA1* and *GSTT1L* promoter were significantly negatively correlated with their mRNA levels (*r* = −0.94, *p* < 0.01, and *r* = −0.87, *p* < 0.05) (Supplementary Figures S2, S3).

### Effect of 5-Azacytidine (5-AZA) on Intramuscular Preadipocytes Differentiation

**FIGURE 8:**
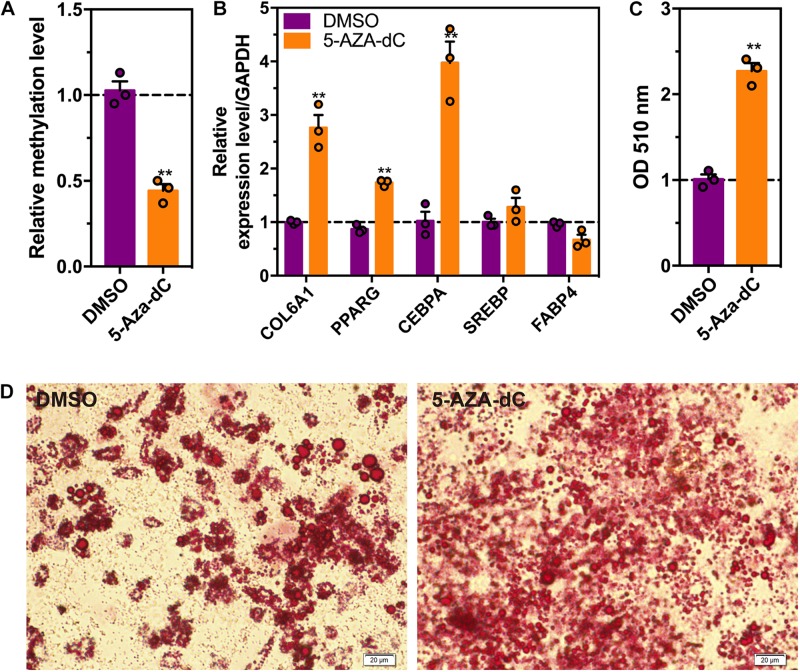
The effects of 5-AZA-dC treatment on intramuscular adipocytes differentiation. **(A)** The DNA methylation level (5 mC) in intramuscular preadipocytes treated with or without 5-AZA-dC (5 μM) for 96 h. **(B)** The relative mRNA levels in intramuscular adipocytes treated with or without 5-AZA-dC (5 μM) for 96 h. **(C,D)**. Oil-red O staining of intramuscular adipocytes treated with or without 5-AZA-dC (5 μM) for 96 h. (*n* = 3). ^∗∗^*p* < 0.01.

To further investigate whether the DNA methylation influence intramuscular adipogenesis, the methylation inhibitor, 5-AZA was used to treat intramuscular preadipocytes. As shown in Figure 8A, the methylation level declined 60% in preadipocytes in the presence of 5-AZA relative to the control cells. Meanwhile, the mRNA levels of *COL6A1* and adipogenic makers, *PPARG* and *CEBPA* were significantly up-regulated after differentiation induction for 48 h in treating with 5-AZA cells (Figure 8B). In addition, Oil Red O staining showed that 5-AZA promoted the intramuscular adipogenesis (Figures 8C,D).

### Chicken COL6A1 Promoted Intramuscular Preadipocytes Proliferation and Differentiation

**FIGURE 9:**
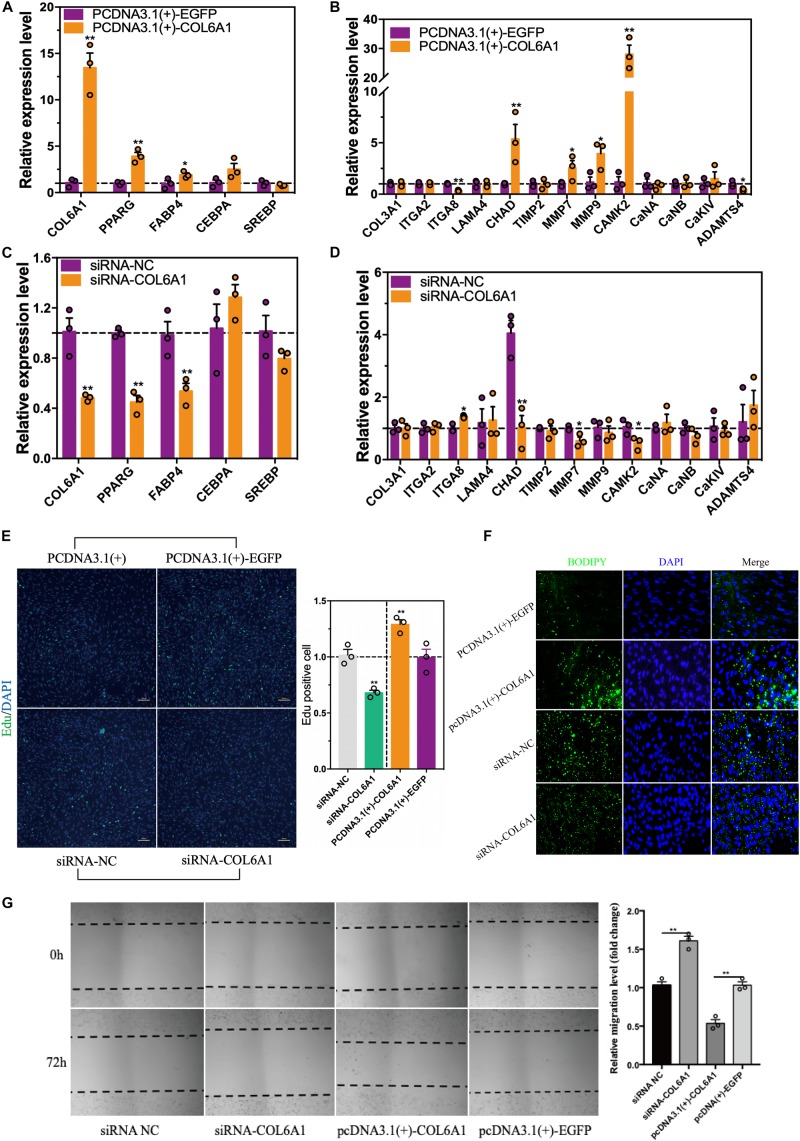
The effects of COL6A1 overexpression and knockdown on cell proliferation, differentiation and migration. **(A)** Overexpressed of COL6A1 promoted the expression of adipogenic differentiation and ECM-related genes **(B)** of intramuscular adipocytes. The relative mRNA levels of genes were detected by qRT-PCR after transfected with plasmid for 48 h. **(C)** Knockdown of COL6A1 suppressed the expression of adipogenic differentiation and ECM-related genes **(D)** of intramuscular adipocytes. The relative mRNA levels of genes were detected by qRT-PCR after transfected with RNA oligos for 24 h. **(E)** COL6A1 promoted intramuscular preadipocytes proliferation. The percentage of EDU positive cells was quantified after transfected with plasmid or RNA oligos. **(F)** COL6A1 accelerated intramuscular preadipocytes differentiation. BODIPY (green) and DAPI (blue) staining of intramuscular adipocytes after transfected with plasmid or RNA oligos. **(G)** COL6A1 promoted intramuscular adipocytes migration. The width of the scratches was measured by microscope after transfected with plasmid or RNA oligos for 72 h (*n* = 3), *p < 0.05, **p < 0.01.

To find out the potential role of *COL6A1* in chicken intramuscular preadipocyte proliferation and differentiation, *COL6A1* overexpression [pcDNA3.1(+)-COL6A1 vs. pcDNA3.1(+)-EGFP] and knockdown (siRNA-NC vs. siRNA-COL6A1) experiments were performed. The mRNA levels of *COL6A1* increased over 13-fold in pcDNA3.1(+)-COL6A1-transfected group compared with control pcDNA3.1(+)-EGFP-transfected group (Figure 9A). Overexpressed *COL6A1* significantly increased the mRNA expression levels of adipogenic makers *PPARG*, *CEBPA*, *FABP4*, and ECM-related genes *CHAD*, *MMP7*, *MMP9*, and *CAMK2* (Figure 9B). In contrast, knockdown the *COL6A1* down-regulated their mRNA expression levels (Figures 9C,D). EDU staining suggested that *COL6A1* promoted intramuscular preadipocytes proliferation (Figure 9E). BODIPY staining showed that overexpressed *COL6A1* significantly promoted the formation of lipid droplet in the intramuscular adipocytes, while decreased lipid droplet formation after RNA interference with *COL6A1* (Figure 9F). Wound healing test suggested that *COL6A1* promoted intramuscular adipocytes migration (Figure 9G).

## Materials and Methods

### Ethics Statement

All animal experiments were conducted with the guidelines of Institutional Animal Care and Use Committee (IACUC) at the Henan Agricultural University (Zhengzhou, Henan, China) (#11-0085).

### Animals and Cells

All of the Gushi chickens were purchased from the Animal Center of Henan Agricultural University (Zhengzhou, Henan, China). Chickens were fed with the same diet *ad libitum* in the same environment. Tissues used for tissues expression profiles were collected and stored at −80°C until use. The breast muscle tissues were used for the IMF preadipocytes isolation according to our previous methods (Zhang et al., 2019).

### DNA Extraction, Library Construction, and Whole Genome Bisulfite Sequencing (WGBS)

Genomic DNA used for WGBS was extracted by an animal genomic DNA kit (Tiangen, China) according to the manufacturer’s instructions. genomic DNA was interrupted into fragments and purified by PCR purification kit. Fragmented DNA was end-repaired, added “A” nucleotide to the 3′end and ligated with methylated adapters. Fragments with adapters were used for bisulfite convertion by a methylation-gold kit (ZYMO, Los Angeles, CA, United States). Furthermore, converted DNA fragments were sequenced by Illumina HiSeq 2500. After removing unknown nucleotides and low-quality reads of raw reads, clean reads were got and used for the downstream analysis.

### Data Analysis

Produced clean reads were mapped to chicken reference genome (GGA_5.0) using the Bismark software (version: 2.90) (Krueger and Andrews, 2011). Then, a methylkit R package (Akalin et al., 2012) was to estimate methylation status and ratio of the CpG sites, promoter region, CpG island region and gene annotation. To get different methylation status in the chicken different genomic regions, methylation levels at 5′-flanking 2 kb regions and gene sequences in different samples were plotted. The RNA-Seq data used in the present study come from our present study (Zhang et al., 2019). The IMF content data used in the present study came from our previous study (Fu et al., 2018).

### Identification of DMRs and Functional Analysis of DMR-Related Genes

The methylation regions with *p* ≤ 0.05 (chi-square test) and the degree of difference methylation >20% were considered as differentially methylated regions (DMRs). DMRs that overlapping with genes body or up or downstream 2 kb of body regions were considered as differentially methylated genes (DMGs). To investigate the functions of the DMGs, GO, and KEGG pathway analysis were conducted in the present study. Fisher’s Exact Test is *p* ≤ 0.05 as threshold.

### Bisulfite Sequencing PCR (BSP)

DNA methylation levels in gene promoters were measured by the Bisulfite sequencing PCR (BSP). Briefly, 200 ng of the chicken preadipocytes and adipocytes genomic DNA was treated with bisulfite. The bisulfite-treated DNA was used for touchdown PCR. BSP primers were designed using the MethPrimer software^1^ (Supplementary Table S1). The PCR products were cloned into the pMD19-T vector (TaKaRa, China) and sequenced by Comate Bioscience Co., Ltd. (Jilin, China). The methylation levels visualizated by MSRcall software^2^.

### Plasmid Construction, RNA Oligos, and Cell Transfection

To construct the overexpressed plasmid of *COL6A1*, the CDS sequence of chicken *COL6A1* was synthesized and cloned into pcDNA3.1(+)-EGFP vector (Invitrogen, United States). Sanger sequencing was performed to confirm the sequence. The siRNAs for *COL6A1* were purchase from GenePharma (Shanghai, China) and transfection with lipofectamine 3000 (Thermo, Shanghai, China). The siRNA-1 of *COL6A1* were: 5′-GGAUGAUGCUGCUAAUGAATT-3′, and 5′-UUCAUUAGCA GCAUCAUCCTT-3′. The siRNA-2 of *COL6A1* were: 5′-GGUC AUCGCCAAAGCUGUUTT-3′, and 5′-AACAGCUUUGGCGA UGACCTT-3′.

### RNA Extraction, cDNA Synthesis and Quantitative Real-Time PCR (qRT-PCR)

Total RNA was isolated by RNAiso Plus (TaKaRa, Dalian, China) following the instruction of manufacturers. The TAKARA PrimeScript^TM^ RT reagent kit (TaKaRa) was used for reverse transcription. The qRT-PCR primers were designed by Primer3plus^3^ (Supplementary Table S1). *GAPDH* was used as internal control to normalized to the expression level of genes. The analysis of genes relative expression levels was using 2^–ΔΔ*Ct*^ method.

### Immunofluorescence Staining

For immunofluorescence, intramuscular adipocytes were fixed with 4% PFA (Beyotime) for 40 min, permeabilized 0.5% Triton X-100 for 10 min, and then blocked with 2% bovine serum albumin (BSA) for 2 h. Following incubated overnight at 4°C with anti-5 mC (Active Motif, 1:200) and anti-5 hmC (Active Motif, 1:200), stained at room temperature for 1 h with Alexa Fluor 488 goat anti-mouse or 594 goat anti-rabbit. The DNA were stained with DAPI (10 μg/mL, Beyotime) for 5 min. The images were captured with fluorescence microscopy (Nikon, Tokyo, Japan). The fluorescence intensity was analyzed by ImageJ software.

### 5-aza-2′-Deoxycytidine (5-Aza-dC) Treatments

After reaching 70–80% confluent, intramuscular preadipocytes were treated with demethylation agent 5-aza-dC (Sigma) (dissoloved in DMSO) at 5 μM for 96 h. DMSO treatment was used as a control. Then cells were induced differentiation for 96 h, then for downstream experiment.

### 5-Methylcytosine (5-mC) Analysis of Genomic DNA

The genomic DNAs were extracted with TIANamp Genomic DNA Kit (TIANGEN) following the instruction of manufacturers. The methylation analysis was performed by the 5 mC DNA ELISA Kit (Zymo Research, United States) following the manufacturer’s instructions. The microplate reader (Thermo Fisher) was used to detect the absorbance at 405 nm.

### 5-Ethynyl-2′-Deoxyuridine (EdU) Assay

After transfection for 48 h, intramuscular adipocytes were incubated at 37°C with 50 μM EdU (RiboBio, China) for 2 h, then cells were fixed with 4% PFA for 30 min and neutralized by 2 mg/mL glycine solution, permeabilized with 0.5% Triton X-100. Then cells were incubated with Apollo Reaction Cocktail (RiboBio, China) for 30 min at room temperature. The DNA was stained with DAPI (Beyotime) for 15 min. The EDU-positive cells were observation with a fluorescence microscope (Nikon, Tokyo, Japan).

### Wound Healing Test

After reached 70–80% confluence, intramuscular preadipocytes were transfected with plasmid or RNA oligos. Subsequently, 10 μL pipette tips were used to generated linear wound. The width of the scratches was measured by microscope (Nikon, Japan) at 0 and 72 h.

### Oil Red O and BODIPY 493/503 Staining

Oil red O staining was performed following our previously method (Zhang et al., 2018). Cells were fixed with10% PFA for 40 min, and then stained with oil red O for 20 min. The dye was extracted by isopropanol incubation for 15 min at room temperature. Quantitative assessment was obtained by microplate reader (Thermo Scientific) at 510 nm. Where indicated, lipids were co-stained by adding BODIPY 493/503 (1 mg/mL, Molecular Probes #D3922) to secondary antibody solution. Cells were washed three times with PBS prior to imaging.

### Statistical Analysis

Statistical analyses were performed using SPSS19 software (SPSS Inc., Chicago, IL, United States). In the present study, the results were presented as mean ± SEM, were subjected to statistical analysis by two-tailed *t*-test. The level of significance was presented as ^∗^*p* < 0.05) and ^∗∗^*p* < 0.01.

## Discussion

IMF content contributes to the meat juiciness and tenderness. Our previous study suggested that the breast muscle of later laying-period hens had higher IMF content than that of juvenile hens, while they exhibited higher global DNA methylation levels (Zhang et al., 2017). Growing numbers of studies demonstrated that DNA methylation played important roles in adipogenesis. Therefore, we speculated that DNA methylation might have great influences on adipogenic differentiation of chicken intramuscular adipocytes *in vitro*.

According to our WGBS data, 60% of mC were found to be existed in the CG context, 0.6% in the CHG context, and 0.7% in the CHH context in the present study. The methylation level at the genome-wide scale was significantly reduced in the mature intramuscular adipocytes. We noticed that the DNA methylation level declined aggressively prior to TSS and gradually rose in the coding region of the chicken genome, which is consistent with previous studies in chicken (Zhang et al., 2017). The exon and intron regions of the chicken genome consisted of a large proportion of the DMRs, a small part of DMRs were belong to the 5′UTR and 3′UTR (Figure 5). The methylation regulation of the intron regions underlying adipocytes differentiation was worth to study in the future.

*DNMT1* mainly involved in maintain methylation (Song et al., 2012), while *DNMT3A*/*3B* mainly involved in the *de novo* DNA methylation (Li et al., 2007; Hervouet et al., 2009). Tet methylcytosine dioxygenases (*TET1*/*2*/*3*) play important roles in elimination of methylation (Williams et al., 2011). qPCR results showed that the mRNA levels of DNA methyltransferases *DNMT1*, *DNMT3A/3B*, and *TET1/2/3* were significantly down-regulated during intramuscular adipocyte differentiation, suggesting that whole-genome DNA demethylation may occur during adipocyte differentiation. The process of adipocyte differentiation requires the initiation of a large number of genes and transcription factors for synergistic expression, which may be related to the differentiation of adipocytes (Mersmann and Ding, 2001). Our previous study found that the hypermethylation in the promoters of *ABCA1, COL6A1*, and *GSTT1L*, thus inhibiting their expression in the later laying-period hens (Zhang et al., 2017). Interestingly, we noticed that they were up-regulated after adipocyte differentiation, suggesting that they may play crucial roles in the differentiation of intramuscular preadipocytes.

*ABCA1* maintains cholesterol homeostasis, regulates lipid metabolism in adipocytes (Schmitz et al., 1999; Schmitz and Langmann, 2005). The DNA methylation level of *ABCA1* affects high density lipoprotein cholesterol (HDLC) levels in patients with familial hypercholesterolemia (Yasuaki et al., 2017). *ABCA1* expression influenced triglyceride metabolism in bovine mammary epithelial cells by regulating the expression of related genes in the lipid metabolism pathway (Chen et al., 2019). *ABCA1* silencing by siRNA also reduce peroxisome proliferator-activated receptor γ (*PPAR*γ) expression and triglyceride content during 3T3-L1 pre-adipocyte differentiation (Cuffe et al., 2018). *ABCA1* is significantly up-regulated after differentiation of 3T3-L1 adipocytes, which is consistent with our study on chicken intramuscular adipocytes (Le et al., 2003). Glutathione S-transferases (*GSTT1*) Glutathione S-transferases influencing the lipid peroxides metabolism during adipocytes differentiation process (Jowsey et al., 2003; Corton et al., 2008). Wang et al. (2009) found that *GSTT1* were upregulated in the adipose tissues of fat line birds compared with lean line birds.

Muscle tenderness is closely related with the content of collagen. The ECM not only affects the development of muscle fibers, but also has an effect on IMF content and tenderness (Cánovas et al., 2010). *COL6A1* gene is involved in cell adhesion and extracellular matrix (ECM). Previous studies suggested that the expression of collagen synthesis related-genes is related to the meat quality of beef (Zhang et al., 2011).

To further investigate the effects of DNA methylation on intramuscular preadipocytes differentiation, we focused on collagen type VI alpha 1 chain gene (*COL6A1*), which is located in the extracellular matrix (ECM) receptor interaction and focal adhesion pathway. With the differentiation of preadipocytes, the lipid droplets gradually fill the cytoplasm, and the cells are easily crushed and ruptured. At this time, the collagen components that act as protective cells in the extracellular matrix are synthesized in large amounts. It is generally believed that DNA methylation of the gene promoter region inhibits gene expression (Lorincz et al., 2004). In our study, we found that the DNA methylation level of *COL6A1* promoter was decreased while the mRNA level was increasing after adipogenic differentiation. The methylation inhibitor, 5-AZA-dC promotes intramuscular adipocytes differentiation by increasing the core adipogenic factors, *PPARG* and *CEBPA*. Furthermore, function loss and gain of experiment of *COL6A1* suggested that DNA methylation can regulate the chicken intramuscular adipocytes differentiation by affecting the expression of ECM-related genes (such as *COL6A1* gene).

## Conclusion

**FIGURE 10:**
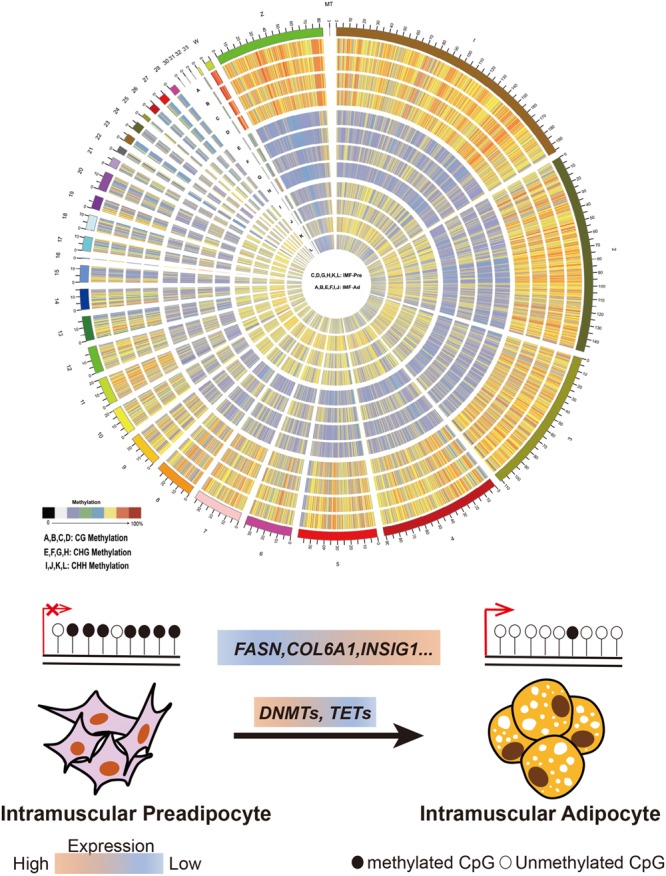
Schema of the epigenetic regulatory mechanism of DNA methylation intramuscular adipogenesis in chicken. The global DNA methylation level decreased with the expression levels of DNA methylasferase (DNMTs, TETs) during intramuscular adipogenic differentiation. Thus, increasing large amount of lipid metabolism and adipocyte differentiation-related genes (such as COL6A1, FASN, and INSIG1, etc.) expression.

In conclusion, our study firstly supplies comprehensive DNA methylation atlas in chicken adipocytes. Integrated DNA methylation with transcriptome, the present study revealed several potential genes (such as *COL6A1*, *FASN*, and *INSIG*, etc.) and pathways related to lipid metabolism and adipocytes differentiation regulated by DNA methylation (Figure 10). Our study will accelerate the study of genome epigenetic mechanism in adipocytes differentiation and IMF deposition in poultry.

## Data Availability Statement

The datasets generated for this study can be found in the PRJNA429489 and PRJNA428933.

## Ethics Statement

The animal study was reviewed and approved by Institutional Animal Care and Use Committee (IACUC).

## Author Contributions

MZ, GS, and XK conceived of and designed the experiments. MZ, DL, YZ, and ZW performed the experiments. MZ, DL, and YZ analyzed the data. ZL, GL, XM, DZ, RH, and RJ contributed reagents, materials, and analysis tools. MZ wrote the manuscript. ZL reviewed the manuscript. All authors approved the final manuscript.

## Conflict of Interest

The authors declare that the research was conducted in the absence of any commercial or financial relationships that could be construed as a potential conflict of interest.

## Footnotes

1 http://www.urogene.org/methprimer/

2 http://www.msrcall.com/MSRcalcalate.aspx

3 http://www.primer3plus.com/cgi-bin/dev/primer3plus.cgi

## Supplementary Material

FIGURE S1 The correlation between the mRNA levels of COL6A1, ABCA1, and GSTT1L and the TG content of intramuscular adipocytes during differentiation process.

FIGURE S2 The DNA Methylation levels of ABCA1 promoter region. **(A,B)** The DNA Methylation levels of ABCA1 promoter region in intramuscular preadipocytes and adipocytes. BSP analyses of the DNA methylation of ABCA1 promoter during intramuscular adipogenic differentiation. **(C)** The correlation between the ABCA1 mRNA levels and DNA methylation levels during intramuscular adipogenic differentiation.

FIGURE S3 The DNA Methylation levels of GSTT1L promoter region. **(A,B)** The DNA Methylation levels of GSTT1L promoter region in intramuscular preadipocytes and adipocytes. BSP analyses of the DNA methylation of GSTT1L promoter during intramuscular adipogenic differentiation. **(C)** The correlation between the GSTT1L mRNA levels and DNA methylation levels during intramuscular adipogenic differentiation.

TABLE S1 Primer sequences for BSP and qRT-PCR.
