# Supplementary figures and images for: The Landscape of DNA Methylation Associated With the Transcriptomic Network of Intramuscular Adipocytes Generates Insight Into Intramuscular Fat Deposition in Chicken
Source: Front Cell Dev Biol. 2020 Apr 2;8:206. doi: 10.3389/fcell.2020.00206 (PMC7142253; doi:10.3389/fcell.2020.00206)

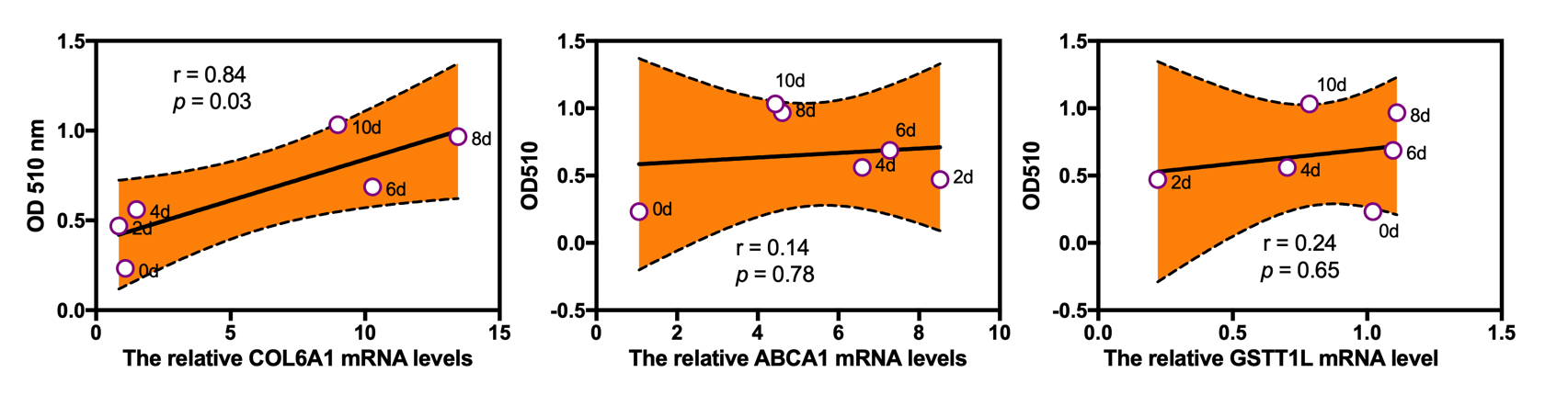

Supplement: FIGURE S1 — The correlation between the mRNA levels of COL6A1, ABCA1, and GSTT1L and the TG content of intramuscular adipocytes during differentiation process. [file Image_1.TIFF]

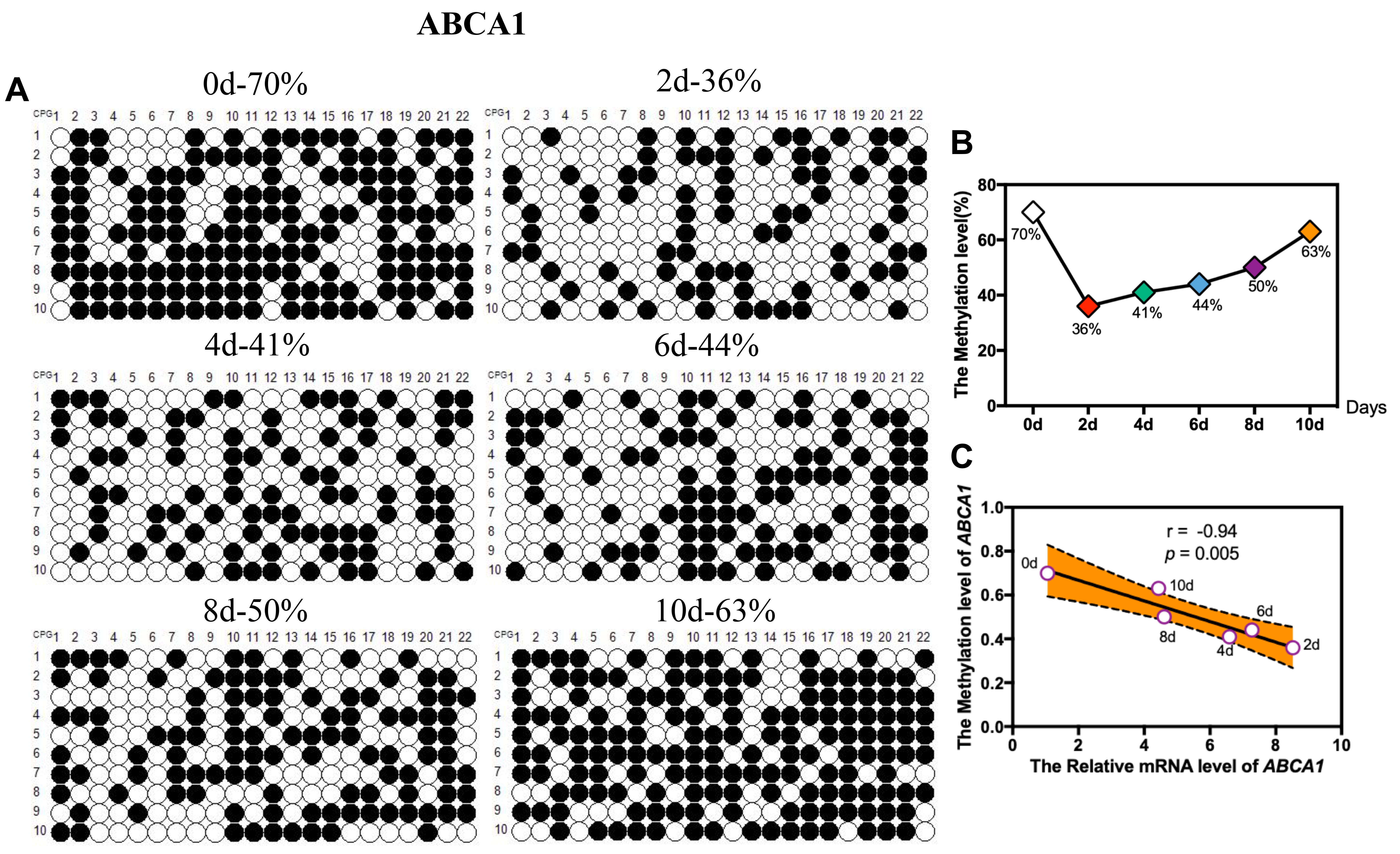

Supplement: FIGURE S2 — The DNA Methylation levels of ABCA1 promoter region. (A,B) The DNA Methylation levels of ABCA1 promoter region in intramuscular preadipocytes and adipocytes. BSP analyses of the DNA methylation of ABCA1 promoter during intramuscular adipogenic differentiation. (C) The correlation between the ABCA1 mRNA levels and DNA methylation levels during intramuscular adipogenic differentiation. [file Image_2.TIF]

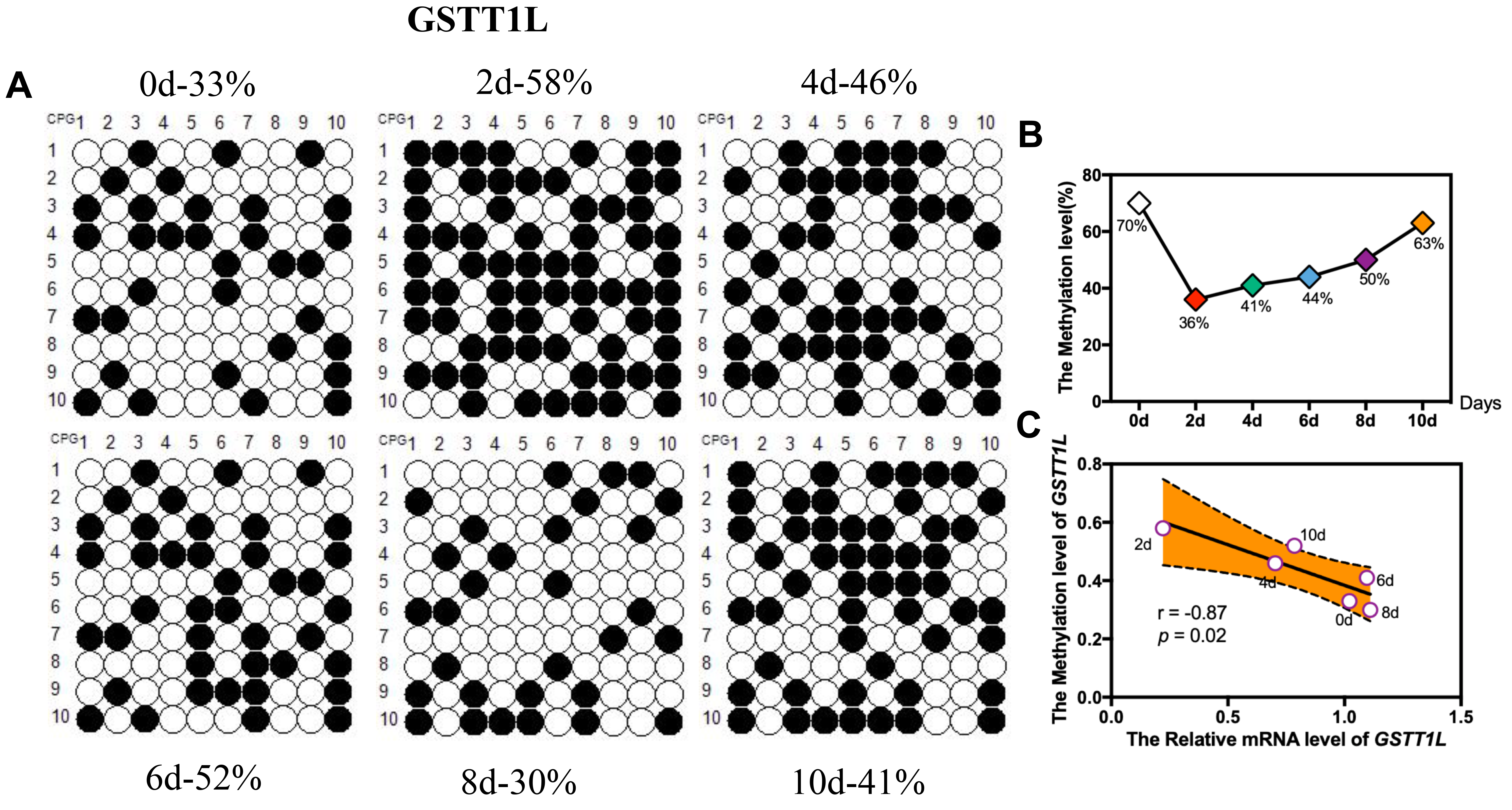

Supplement: FIGURE S3 — The DNA Methylation levels of GSTT1L promoter region. (A,B) The DNA Methylation levels of GSTT1L promoter region in intramuscular preadipocytes and adipocytes. BSP analyses of the DNA methylation of GSTT1L promoter during intramuscular adipogenic differentiation. (C) The correlation between the GSTT1L mRNA levels and DNA methylation levels during intramuscular adipogenic differentiation. [file Image_3.TIF]
