# Supplementary material for: The Landscape of DNA Methylation Associated With the Transcriptomic Network of Intramuscular Adipocytes Generates Insight Into Intramuscular Fat Deposition in Chicken
Source: Front Cell Dev Biol. 2020 Apr 2;8:206. doi: 10.3389/fcell.2020.00206 (PMC7142253; doi:10.3389/fcell.2020.00206)
Supplement: TABLE S1 — Primer sequences for BSP and qRT-PCR. [file Table_1.DOCX]

Table S1 Primer sequences for BSP and qRT-PCR.

| Genes | Primer sequences(5’-3’) | Product size(bp) | Tm |
| --- | --- | --- | --- |
| COL6A1-F_a_ | TGTGTTGGATATTTTTGAAAGTGT | 228 | —— |
| COL6A1-R_a_ | CTATTCTTCAACTCAACCCTACC |  |  |
| GSTT1L-F_a_ | GTAGTATGGGGTTGGAGTTGTATTT | 179 | —— |
| GSTT1L-R_a_ | CCTCAATACAAACACTTTCATCAAC |  |  |
| ABCA1-F_a_ | GGATTYGTGAGTGGTAGAGTT | 260 | —— |
| ABCA1-R_a_ | ACCATCTTCCCAATCAAACTAT |  |  |
| COL6A1-F | GAAAGCACCCACACTGACT | 200 | 60 |
| COL6A1-R | TGGCGACAGAGAACACTTTG |  |  |
| GSTT1L-F | GTGCTACCGAGGAGCTGAAC | 232 | 60 |
| GSTT1L-R | TCGTGTGCTTCTTGGAACAG |  |  |
| ABCA1-F | CTTCGGGATTTCCTGATTGA | 205 | 60 |
| ABCA1-R | CCACTGAGCGATTTTGGATT |  |  |
| GAPDH-F | AGAACATCATCCCAGCGT | 184 | 60 |
| GAPDH-R | AGCCTTCACTACCCTCTTG |  |  |
| DNMT1-F | TGTCCATCTTCGACGCCAAC | 176 | 60 |
| DNMT1-R | CATAGATGGGCTTCACGGCA |  |  |
| DNMT3A-F | GGGTGAGCGACAAAAGGGAC | 234 | 60 |
| DNMT3A-R | TGGAGTTGGAGCGAGTGGTG |  |  |
| DNMT3B-F | ACCAGCCAAGAGGAGACCCA | 269 | 60 |
| DNMT3B-R | TGGCGAGCGAGAGGTCATTA |  |  |
| TET1-F | GACTCAGGGTGTTCTCCAGC | 213 | 60 |
| TET1-R | CACAGCAGCAACACTTGGTC |  |  |
| TET2-F | TCGAGTACGAACACAGAGCG | 147 | 60 |
| TET2-R | TGCAAACCAGTGTACTCCCG |  |  |
| TET3-F | CAGAATGCAGGGTATGGCGT | 156 | 60 |
| TET3-R | TCATGCTGTAAGGGTCGGAG |  |  |
| PPARG-F | GTGCAATCAAAATGGAGCC | 170 | 60 |
| PPARG-R | CTTACAACCTTCACATGCAT |  |  |
| FABP4-F | ATGTGCGACCAGTTTGT | 143 | 60 |
| FABP4-R | TCACCATTGATGCTGATAG |  |  |
| CEBPA-F | TTCTACGAGGTCGATTCCCG | 96 | 60 |
| CEBPA-R | AGCCTCTCTGTAGCCGTAG |  |  |
| SREBP-F | GGTCCGGGCCATGTTGA | 175 | 60 |
| SREBP-R | CAGGTTGGTGCGGGTGA |  |  |
| COL3A1-F | TGCTTGTGGCTGAGTTCTGT | 214 | 60 |
| COL3A1-R | CGTCCTGTTGTGCCAAAAT |  |  |
| ITGA2-F | GCCTACAATGTTGGGCTCCT | 134 | 60 |
| ITGA2-R | GGATAGCCACTCCAGGGTGA |  |  |
| ITGA8-F | CCTCAGTAACGGAACAGGCT | 238 | 60 |
| ITGA8-R | AGTGCCTTGTTGTTGCGTTC |  |  |
| LAMA4-F | AGCACCTCCAGACTTCAAGC | 221 | 60 |
| LAMA4-R | CCACAGCATAGCCAGAACCA |  |  |
| CHAD-F | GCAGTCCATCAGAGACACCC | 96 | 60 |
| CHAD-R | GCCATACTGGGTCGTGCTTA |  |  |
| TIMP2-F | CCCCATCAAGCGAATCCAGT | 130 | 60 |
| TIMP2-R | CTTGCCTCCTGTGTCCAGAG |  |  |
| MMP7-F | CGCTGCGCTTCAAAAGAGTT | 134 | 60 |
| MMP7-R | CCCCTCTCCAGGCTGAAAAG |  |  |
| MMP9-F | GGGGTTTCCTCACCTTCGAG | 222 | 60 |
| MMP9-R | ACCCATCACCATGCTCTTGG |  |  |
| CAMK2-F | CCAAGGGGAACAACAGGC | 385 | 60 |
| CAMK2-R | CTTCAAGCAATCTACCGTCT |  |  |
| CaNA-F | TTCAACTGCTCCCCTCAT | 151 | 60 |
| CaNA-R | AACCATCTTCTTCTGTCCCT |  |  |
| CaNB-F | TGGAGGACGACAGACCC | 245 | 60 |
| CaNB-R | CAGGCAAGACATAAGTGAGTAA |  |  |
| CaKIV-F | GCAGGCAGAAAGGGAC | 105 | 60 |
| CaKIV-R | GTGAAAGGCGAAGAAGG |  |  |
| ADAMTS4-F | TTGGAGAGGGGATGGAGAGG | 102 | 60 |
| ADAMTS4-R | ACTCTGACCTCTGGGGATCC |  |  |

^a^Primers for BSP.
